# Effects of Processing Parameters on the Synthesis of (K_**0.5**_Na_**0.5**_)NbO_**3**_ Nanopowders by Reactive High-Energy Ball Milling Method

**DOI:** 10.1155/2014/203047

**Published:** 2014-01-27

**Authors:** Nguyen Duc Van

**Affiliations:** Institute of Materials Science, Vietnam Academy of Science and Technology, 18 Hoang Quoc Viet, Cau Giay, Hanoi 10000, Vietnam

## Abstract

The effects of ball milling parameters, namely, the ball-to-powder mass ratio and milling speed, on the synthesis of (K_0.5_Na_0.5_)NbO_3_ nanopowders by high-energy ball milling method from a stoichiometric mixture containing Na_2_CO_3_, K_2_CO_3_, and Nb_2_O_5_ were investigated in this paper. The results indicated that the single crystalline phase of (K_0.5_Na_0.5_)NbO_3_ was received in as-milled samples synthesized using optimized ball-to-powder mass ratio of 35 : 1 and at a milling speed of 600 rpm for 5 h. In the optimized as-milled samples, no remaining alkali carbonates that can provide the volatilizable potassium-containing species were found and (K_0.5_Na_0.5_)NbO_3_ nanopowders were readily obtained via the formation of an intermediate carbonato complex. This complex was mostly transformed into (K_0.5_Na_0.5_)NbO_3_ at temperature as low as 350°C and its existence was no longer detected at spectroscopic level when calcination temperature crossed over 700°C.

## 1. Introduction

Nowadays, with the tendency towards the sustainable and eco-friendly development, lead-containing compounds in general and commonly-used Pb (Zr,Ti)O_3_-based piezoelectric materials with about 60 wt.% of lead in particular are restricted to use and are going to be expelled due to their high toxicity [1, 2]. For (K_0.5_Na_0.5_)NbO_3_-based material, one of the most perspective lead-free piezoelectric candidates, the finding out of alternatives to the conventional solid-state method attracted many researchers to overcome its drawback of providing nonstoichiometric sintered product with low density [3–5]. This drawback originated from the phase instability of alkali niobate at high temperature and the high volatility of potassium-containing species existed in reaction mixture [6, 7]. To date, many preparative techniques like wet chemistry methods (hydrothermal, sol-gel,…) and modified solid-state reaction methods have been applied as preparative alternatives to the conventional solid-state reaction [8–11]. Among these applied techniques, sol-gel and mechanochemical-assisted solid-state reaction methods have drawn a lot of interest due to the fact that they can provide potassium sodium niobate nanopowders. These nanosized powders possess a high surface area and stored energy for solid-state densification at low temperatures [11] and, as a result, the sinterability of samples can be improved. However, the formation of hydrated carbonated phases that can be only decomposed at high temperatures was detected for samples synthesized by sol-gel method [9, 10]. By using mechanochemical-assisted solid-state reaction method, the calcination temperature required for decomposition of carbonates that existed in mechanochemical activated mixture was still relatively high that might lead to a certain loss of volatilizable potassium-containing species if alkali carbonates were used as raw materials. Although the direct synthesis procedure of (K_0.5_Na_0.5_)NbO_3_ for obtaining nanopowders readily by mechanochemical method was published by us [12] for the first time recently, the effects of the ball-to-powder mass ratio and milling speed on the synthesis of (K_0.5_Na_0.5_)NbO_3_ nanopowders have been not yet studied. In addition, for the direct synthesis of (K_0.5_Na_0.5_)NbO_3_ as well as NaNbO_3_ [13] or for the mechanochemical-assisted solid-state synthesis of (K,Na,Li)(Nb,Ta,Sb)O_3_ [11], an intermediate carbonato complex was found during the processing, but its thermal decomposition behaviour had not been reported to date.

The effects of ball-to-powder mass ratio and milling speed on the synthesis of (K_0.5_Na_0.5_)NbO_3_ nanopowders by high-energy ball milling method using Na_2_CO_3_, K_2_CO_3_, and Nb_2_O_5_ as starting materials were described in detail in this paper. In addition, the thermal decomposition of the carbonato complex as an intermediate species during the reactive high-energy ball milling process was also investigated.

## 2. Materials and Methods

For high-energy ball milling synthesis of (K_0.5_Na_0.5_)NbO_3_, all analytical grade K_2_CO_3_, Na_2_CO_3_, and Nb_2_O_5_, which were purchased from Aldrich (Germany), were dried at 200°C for 2 h prior to use in order to remove moisture. Stoichiometric mixtures of these raw materials were placed in a 125 mL stainless-steel vial of a Fritsch Pulverisette 6 planetary mill. The rotational speed was changed from 300 to 600 rpm. Different numbers of stainless-steel milling balls with diameters of 10 and 20 mm were used depending on the ball-to-powder weight ratio ranging from 25/1 to 40/1. For investigation of thermal decomposition of intermediate carbonate complex, as-milled samples synthesized at optimized conditions were calcined at temperatures ranging from 300 to 1000°C for 3 h. X-ray diffraction diagrams of obtained samples were recorded using Siemens D-5000 diffractometer (Siemens, Germany) with CuK_*α*_ radiation. For the determination of lattice parameters, Si (Aldrich, Germany) was used as an internal standard. The synthesized samples were also characterized by field-emission scanning electron microscopy (FE-SEM, Hitachi S 4800 microscope, Japan), Fourier transform infra-red spectroscopy (FTIR, GX-Perkin-Elmer, USA), and thermal analysis (Setaram Labsys Evo thermal analyser, France).

## 3. Results and Discussion 

### 3.1. Effects of Ball-to-Powder Weight Ratio on the Phase Formation of (K_0.5_Na_0.5_)NbO_3_

**Figure 1:**
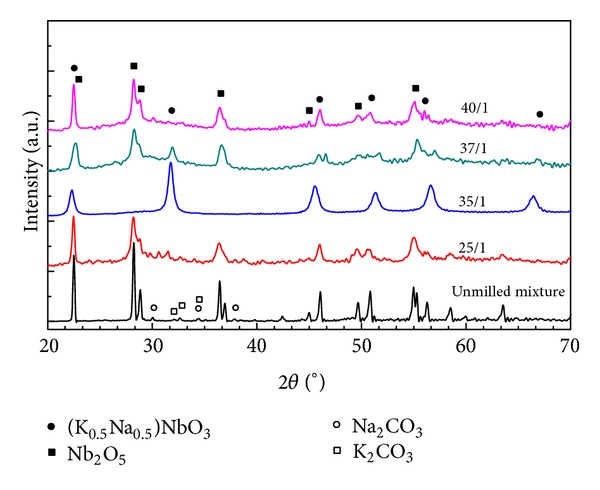
X-ray diffraction diagrams of unmilled mixture and as-milled (K_0.5_Na_0.5_)NbO_3_ prepared mechanochemically at 600 rpm for 5 h with different ball-to-powder mass ratios of 25/1, 35/1, 37/1, and 40/1.

**Figure 2:**
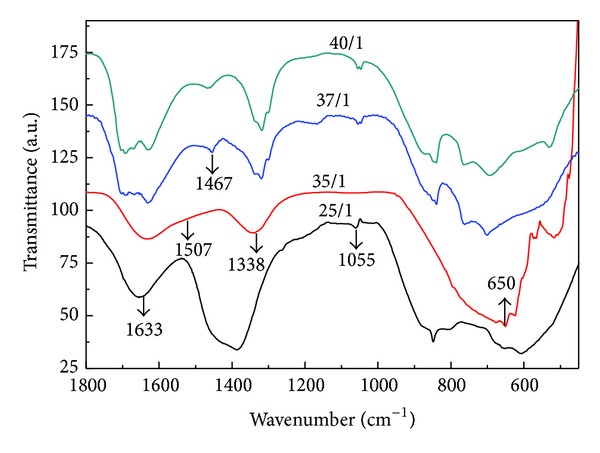
FTIR spectra of as-milled (K_0.5_Na_0.5_)NbO_3_ prepared mechanochemically at 600 rpm for 5 h with different ball-to-powder mass ratios of 25/1, 35/1, 37/1, and 40/1.

**Figure 3:**
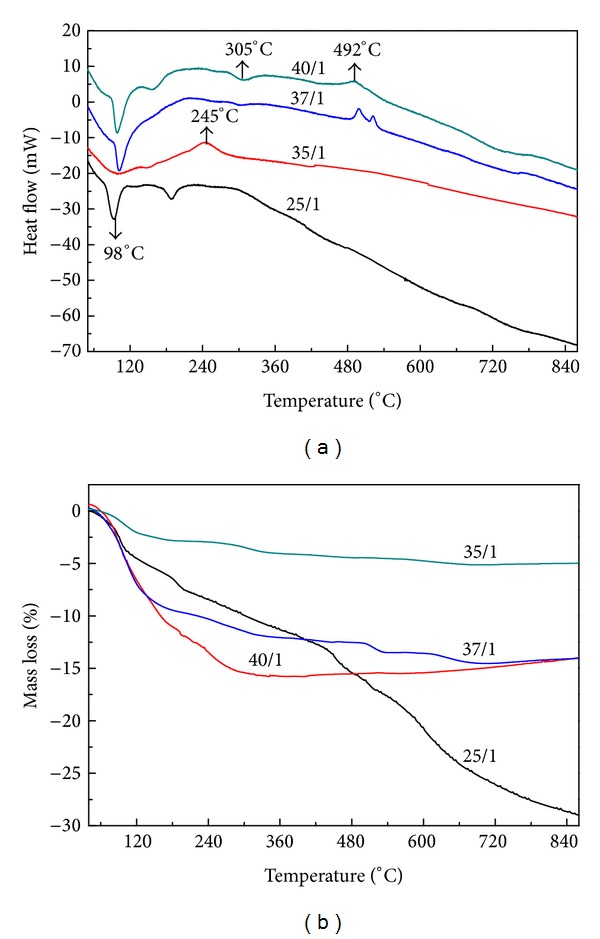
(a) DTA and (b) TG curves of as-milled (K_0.5_Na_0.5_)NbO_3_ prepared mechanochemically at 600 rpm for 5 h with different ball-to-powder mass ratios of 25/1, 35/1, 37/1, and 40/1.

X-ray diffraction (XRD) diagrams of an unmilled mixture containing K_2_CO_3_, Na_2_CO_3_, Nb_2_O_5_, and as-milled (K_0.5_Na_0.5_)NbO_3_ samples synthesized at rotational speed of 600 rpm for 5 h with different ball-to-powder weight ratios (BPRs) of 25/1, 35/1, 37/1, and 40/1 were shown in Figure 1. By using the ball-to-powder weight ratio of 25/1, all observable broaden peaks in XRD diagram can be assigned to Nb_2_O_5_ (PDF card number 27-1003) only. It can be explained that the milling energy produced during the ball milling process was insufficient to activate Nb_2_O_5_ to react with alkali carbonates at atomic scale. Instead, this energy was mainly supplied for breaking down bulk Nb_2_O_5_ into nanocrystals and for the amorphization of alkali carbonates, similar to the case of NaNbO_3_ reported in [13]. With the BPR of 35/1, a single crystalline phase of (K_0.5_Na_0.5_)NbO_3_ was obtained directly and uniquely without any other impurity. With the selected milling parameters, the generated milling energy was suggested to be enough for all reactants to react simultaneously and completely to form the desired (K_0.5_Na_0.5_)NbO_3_ crystalline phase. This alkali niobate phase was still detected together with the remaining Nb_2_O_5_ in the as-milled sample by increasing the ball-to-powder weight ratio to 37/1. When the BPR reached 40/1, however, only peaks of Nb_2_O_5_ crystalline phase existed in XRD diagram. Thus, the alkali niobate crystaline phase was only partially formed by selecting the ball-to-powder weight ratio of 37/1 that slightly differed from 35/1 and was completely absent when this value was large enough (40/1). It can be explained that when the ball-to-powder weight ratio crossed over 35/1, Nb_2_O_5_ was assumed to be activated, but K_2_CO_3_ and Na_2_CO_3_ were probably already well amorphized. As a result, these alkali carbonates undergo partially or did not undergo the mechanochemical reaction at all with niobium pentoxide to form alkali niobate phase. Another reason for the above-mentioned phenomenon is that the increased milling energy with the BPR might lead to the amorphization of milling mixture components by creating a more strain and a more defect concentration [14, 15]. Thus, with the presence of large strain and defect concentration, a part or all freshly nucleated crystalline alkali niobates will transform into amorphous state instead of growing up once high ball-to-powder weight ratios (37/1 and 40/1) were chosen. The infra-red spectra of as-milled (K_0.5_Na_0.5_)NbO_3_ samples synthesized at the rotational speed of 600 rpm for 5 h with different BPRs of 25/1, 35/1, 37/1, and 40/1 were shown in Figure 2. In all these spectra, a broad band located at 650 cm^−1^ belongs to symmetrical stretching mode of NbO_6_ octahedra. One can realize that the absorption band at 1467 cm^−1^ assigning to the asymmetrical C–O stretching vibration (*ν*_3_) of free CO_3_^2−^ was absent only for the sample synthesized mechanochemically with the BPR of 35/1. This implied that, with the selected preparative condition, no alkali carbonates were left in the milled product while were still detected in FTIR spectra of samples synthesized with other investigated BPRs. The damping of *ν*_3_ vibration was related to its splitting into three new bands at 1633, 1507, and 1338 cm^−1^. It is worth noting that the in-plane bending vibration of adsorbed water also contributed to the band at 1633 cm^−1^. For samples synthesized with the BPRs of 25/1, 37/1, and 40/1, only two bands at 1633 and 1338 cm^−1^ were detected, while all three bands occurred for the case with the BPR of 35/1. In addition, another new band belonging to the symmetrical C–O stretching vibration at 1055 cm^−1^ (*ν*_1_) that reported to be IR-inactive for the free CO_3_^2−^ of alkali carbonates was also found in all spectra [13, 16, 17]. The above-mentioned four new bands were evidence for the lowering of the symmetry of the CO_3_^2−^ anion via the formation of an amorphous intermediate carbonato complex between CO_3_^2−^ and Nb^5+^ ions. These obtained results are in good agreement with those published previously [13, 16]. The thermal analysis diagrams of samples synthesized with the BPRs of 25/1, 35/1, 37/1, and 40/1 were shown in Figure 3. In TG curve of the as-milled sample synthesized with the BPRs of 25/1, the weight loss of 28.65% up to 900°C associated with the decomposition of carbonates, carbonato complex and the volatilization of potassium-containing species was detected. For samples synthesized with the BPRs of 37/1 and 40/1, the remaining activated carbonates and carbonate complex underwent multistep thermal decomposition with the total weight loss of about 15% by heating up to 700°C, similar to that of mechanochemical-assisted solid-state reaction [11]. It can be suggested that the produced milling energy was not enough to activate all alkali carbonates although the formation of carbonato complex was already evident by using the BPRs of 25/1, while all unreacted carbonates were activated and their thermal decomposition temperatures were lower for the BPRs of 37/1 and 40/1. By using the ball-to-powder mass ratio of 35/1, however, the weight loss of 5.3% was completed at temperature as low as 350°C and no further change was observed in TG curve. This small total weight loss and low decomposition temperature were suggested to be caused only by the transformation of carbonato complex into (K_0.5_Na_0.5_)NbO_3_ and were much lower than those obtained for as-milled samples synthesized with other investigated BPRs. It can be assumed that a larger amount of CO_2_ might be already released from reaction between alkali carbonates and niobium pentoxide to form carbonato complex during ball milling process and this led to the significant lowering in total weight loss. Thus, from above results, it is obvious that, with this optimized ball-to-powder, the volatilization of potassium-containing species produced from the decomposition of remained alkali carbonates during the calcination step that often required for mechanochemical-assisted solid-state reaction procedures can be avoided [11].

### 3.2. Effects of Ball Milling Speed on the Phase Formation of (K_0.5_Na_0.5_)NbO_3_

**Figure 4:**
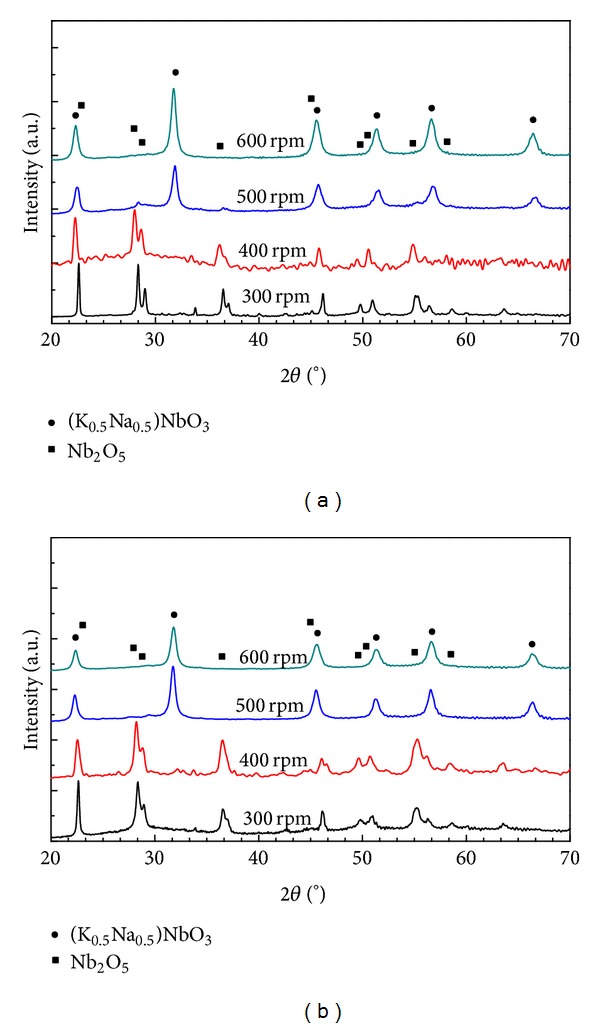
X-ray diffraction diagrams of as-milled (K_0.5_Na_0.5_)NbO_3_ prepared mechanochemically for (a) 5 h and (b) 10 h with the ball-to-powder mass ratio of 35/1 at 300, 400, 500, and 600 rpm.

Figure 4(a) presents XRD diagrams of as-milled samples prepared at different rotational speeds of 300, 400, 500, and 600 rpm with the ball-to-powder weight ratio of 35/1 for 5 h. For two rotational speeds of 300 and 400 rpm, only crystalline phase of Nb_2_O_5_ (PDF card number 27-1003) was found in as-milled samples. By increasing the rotational speed to 500 rpm, (K_0.5_Na_0.5_)NbO_3_ phase occurred while diffraction peaks of Nb_2_O_5_ phase still presented. With the rotational speed of 600 rpm, there are only peaks of the crystalline phase of (K_0.5_Na_0.5_)NbO_3_ in XRD diagram. Thus, for the investigated milling time of 5 h, the milling energy that was generated during ball milling process increased with the rotational speed and finally became enough for all starting materials to be involved in the mechanochemical reaction simultaneously and stoichiometrically to obtain single crystalline phase of (K_0.5_Na_0.5_)NbO_3_ with the rotational speed of 600 rpm. When the milling time was increased to 10 h, the single crystalline phase of (K_0.5_Na_0.5_)NbO_3_ existed not only for the sample synthesized with the rotational speed of 600 rpm but also for that with 500 rpm (Figure 4(b)). For each of the two rotational speeds of 300 and 400 rpm, however, there were only peaks of Nb_2_O_5_ phase in XRD diagrams. From these results, it can be proposed that, with these two low rotational speeds, the generated milling energy was thermodynamically insufficient for high-energy ball milling reaction between Nb_2_O_5_ and alkali carbonates, similar to those of previous works on the synthesis of KNbO_3_ and (K_0.5_Na_0.5_)NbO_3_ [16, 18].

### 3.3. Thermal Decomposition of Intermediate Carbonato Complex

**Figure 5:**
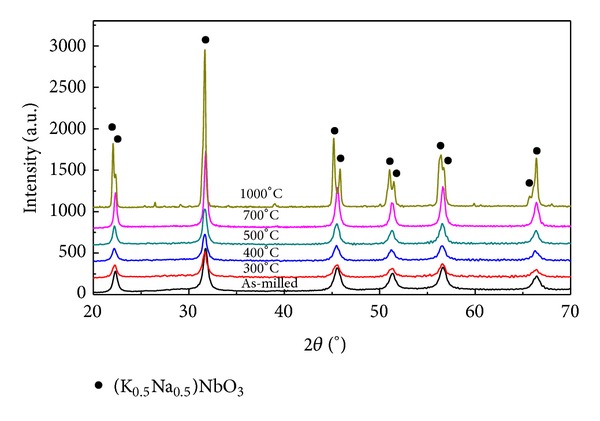
X-ray diffraction diagrams of as-milled (K_0.5_Na_0.5_)NbO_3_ and samples calcined at 300, 400, 500, 700, and 1000°C for 3 h.

**Figure 6:**
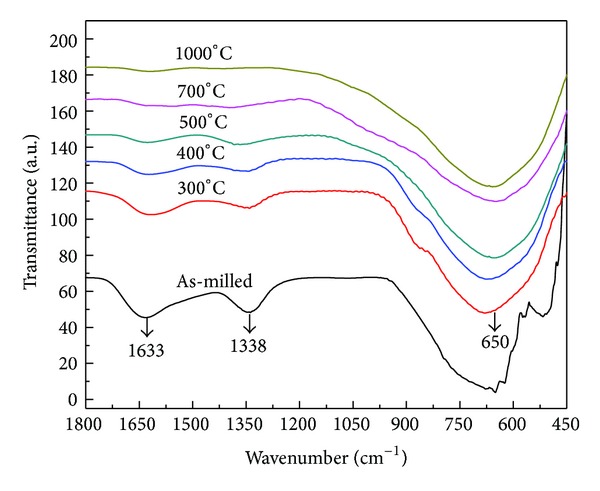
FTIR spectra of as-milled (K_0.5_Na_0.5_)NbO_3_ and samples calcined at 300, 400, 500, 700, and 1000°C for 3 h.

**Figure 7:**
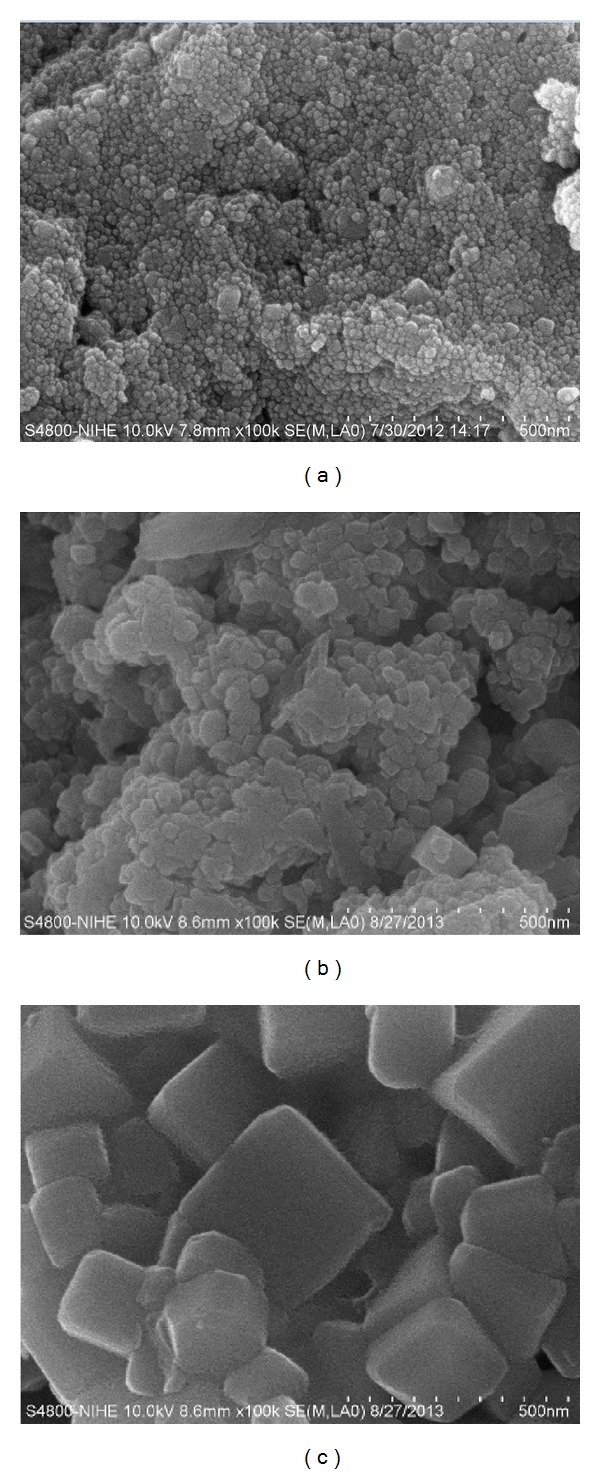
FE-SEM images of (a) as-milled (K_0.5_Na_0.5_)NbO_3_ and samples calcined at (b) 700 and (c) 1000°C for 3 h.

In order to study the thermal decomposition of intermediate carbonato complex, as-milled samples synthesized at optimized conditions were calcined at 300, 400, 500, 700, and 1000°C for 3 h. For all studied samples, only crystalline phase of (K_0.5_Na_0.5_)NbO_3_ was observed in XRD diagrams as shown in Figure 5. As mentioned above, however, the amorphous carbonato complex still existed in samples calcined at low temperatures due to the fact that it was mostly transformed into (K_0.5_Na_0.5_)NbO_3_ phase only when heating temperature reached the value of 350°C. By increasing the calcination temperature, two bands at 1633 and 1338 cm^−1^ that are characteristics for this complex were detectable in FTIR spectra for samples calcined at 400 and 500°C (Figure 6). Its spectroscopic amount was removed only when the samples were calcined over 700°C with the disappearance of the absorption band at 1338 cm^−1^. Although during this calcination the grain growth was developed, carbonato complex-free (K_0.5_Na_0.5_)NbO_3_ nanopowders were achieved with the average grain size of 40 nm for samples calcined at 700°C (Figure 7). From these results, in comparison to mechanochemical-assisted solid-state reaction [11] and sol-gel methods [9, 10], one can see that our reactive high-energy ball milling procedure provided (K_0.5_Na_0.5_)NbO_3_ nanopowders directly and at a large scale without the appearance of any remained alkali carbonates and hydrated carbonated phases.

## 4. Conclusion

The effects of the ball-to-powder mass ratio and milling speed on the synthesis of (K_0.5_Na_0.5_)NbO_3_ nanopowders by reactive high-energy ball milling method using Na_2_CO_3_, K_2_CO_3_, and Nb_2_O_5_ as starting materials were systematically investigated. By selecting the rotational speed of 600 rpm with the ball-to-powder weight ratio of 35/1 after 5 h of milling time as optimized synthesis parameters, the volatilization of potassium-containing species can be avoided and (K_0.5_Na_0.5_)NbO_3_ nanopowders were readily obtained at a large scale via the formation of an intermediate carbonato complex. This complex was mostly transformed into (K_0.5_Na_0.5_)NbO_3_ at temperature as low as 350°C and the carbonato complex-free (K_0.5_Na_0.5_)NbO_3_ nanopowders were obtained with the average grain size of 40 nm for samples calcined at 700°C.
